# Novel Associations of *VKORC1* Variants with Higher Acenocoumarol Requirements

**DOI:** 10.1371/journal.pone.0064469

**Published:** 2013-05-17

**Authors:** Ana Isabel Anton, Juan J. Cerezo-Manchado, Jose Padilla, Virginia Perez-Andreu, Javier Corral, Vicente Vicente, Vanessa Roldan, Rocio Gonzalez-Conejero

**Affiliations:** Centro Regional de Hemodonación and Morales Meseguer Hospital, University of Murcia, Murcia, Spain; University of Bristol, United Kingdom

## Abstract

**Background:**

Algorithms combining both clinical and genetic data have been developed to improve oral anticoagulant therapy. Three polymorphisms in two genes, *VKORC1* and *CYP2C9*, are the main coumarin dose determinants and no additional polymorphisms of any relevant pharmacogenetic importance have been identified.

**Objectives:**

To identify new genetic variations in *VKORC1* with relevance for oral anticoagulant therapy.

**Methods and Results:**

3949 consecutive patients taking acenocoumarol were genotyped for the *VKORC1* rs9923231 and CY2C9* polymorphisms. Of these, 145 patients with a dose outside the expected range for the genetic profile determined by these polymorphisms were selected. Clinical factors explained the phenotype in 88 patients. In the remaining 57 patients, all with higher doses than expected, we sequenced the *VKORC1* gene and genetic changes were identified in 14 patients. Four patients carried *VKORC1* variants previously related to high coumarin doses (L128R, N = 1 and D36Y, N =  3).Three polymorphisms were also detected: rs17878544 (N = 5), rs55894764 (N = 4) and rs7200749 (N = 2) which was in linkage disequilibrium with rs17878544. Finally, 2 patients had lost the rs9923231/rs9934438 linkage. The prevalence of these variations was higher in these patients than in the whole sample. Multivariate linear regression analysis revealed that only D36Y and rs55894764 variants significantly affect the dose, although the improvement in the prediction model is small (from 39% to 40%).

**Conclusion:**

Our strategy identified novel associations of *VKORC1* variants with higher acenocoumarol doses albeit with a low effect size. Further studies are necessary to test their influence on the *VKORC1* function and the cost/benefit of their inclusion in pharmacogenetic algorithms.

## Introduction

The prevention of stroke, deep vein thrombosis, pulmonary embolism or deleterious coronary malfunctions by means of coumarins is one of the most frequently used clinical practices in the world. However, the inherent risk of serious side effects and the wide inter-patient variation in therapeutic dose [1] have highlighted the need to better predict the most suitable dose before starting treatment [2]. The huge variability in the required dose of coumarins to achieve a narrow therapeutic index has been attributed to both clinical factors (mainly age, gender and body mass index) and DNA variants in enzymes, whose activities impact the pharmacokinetic and/or pharmacodynamic of coumarins [3].

In this framework several genome-wide association studies (GWAS) have recently been conducted to detect DNA variants which influence warfarin dosing [4]–[6]. The results of these GWAS have provided a sufficient basis to consider three genetic factors as the strongest markers of coumarin pharmacogenetics: common polymorphisms (SNPs) in *VKORC1*, the target of the drugs, explain up to ∼30% of variance in coumarin dose, while two SNPs in *CYP2C9* (the main metabolizer complex) predict ∼10%. With less impact, rs2108622 in *CYP4F2* determines approximately 1.5% of variance in the coumarin dose and non-genetic factors jointly account for another ∼15% [7].

In an attempt to personalize warfarin doses several models have been developed by incorporating both sets of factors into algorithms that explain as much as 50% of dose variability in Caucasians [7], [8]. Such a percentage has been robustly and widely replicated in several studies, making this area one of the most successful applications of pharmacogenetics to date [9], [10], which is why the US FDA updated the warfarin label in 2007 by including genetic data [11]. However, the issue is still conflictive as the American College of Chest Physicians in the last evidence-based clinical practice guideline about antithrombotic therapy and the prevention of thrombosis does not recommend the routine use of pharmacogenetic testing for guiding doses of coumarins in patients initiating anti-vitamin K therapy (Grade 1B) [12].

Regarding other coumarins used in Europe, e.g. phenprocoumon and acenocoumarol, the main dose determinants are quite similar to warfarin [13], [14] and dosing algorithms have also been developed for them [15], [16]. The application of these models should confirm or not whether the genetic forecasting is of use not only clinically but also economically.

Unfortunately, a still undetermined proportion of patients escape genetic forecasting due to unknown factors, genetic mutations in *VKORC1* and/or *CYP2C9*, or interactions with drugs, food and/or herbs [7]. Accordingly, efforts are also ongoing to identify the additional genetic and clinical factors that would decide the individual coumarin dose necessary. In this work we aim to identify hidden genetic factors involved in deviations from theoretical forecasts as defined by the three most decisive factors, *VKORC1* and *CYP2C9* genotypes.

## Materials and Methods

### Patients

A retrospective study recruited 3949 consecutive Caucasian patients taking acenocoumarol from the Antithrombotic Therapy Unit of the Morales Meseguer University Hospital (Murcia, Spain). Patients were eligible to take part in the study if aged 18 years or over and were steadily anticoagulated. A stable period was defined as a period of at least 3 weeks with three or more consecutive INR measurements with <10% change within the target range in the acenocoumarol dose. Exclusion criteria were a diagnosis of cancer, alcohol or drug abuse, acenocoumarol allergy/intolerance, terminal disease,known or suspected pregnancy and intake of amiodarone, antifungal drugs, anti-platelet drugs and/or statins.

Clinical and demographical data were obtained from the medical records and the database: 1947 males (49%); median age, 74±10 years; median body mass index (BMI), 29.5±7.4. Most patients (90%) were anticoagulated with an INR target range of 2.0–3.0 and 10% of them had an INR target range of 2.5–3.5.

All subjects gave their informed consent according to the Helsinki Declaration, and the study was approved by the Ethical Committee of the Morales Meseguer Hospital.

### Study Design

**Figure 1:**
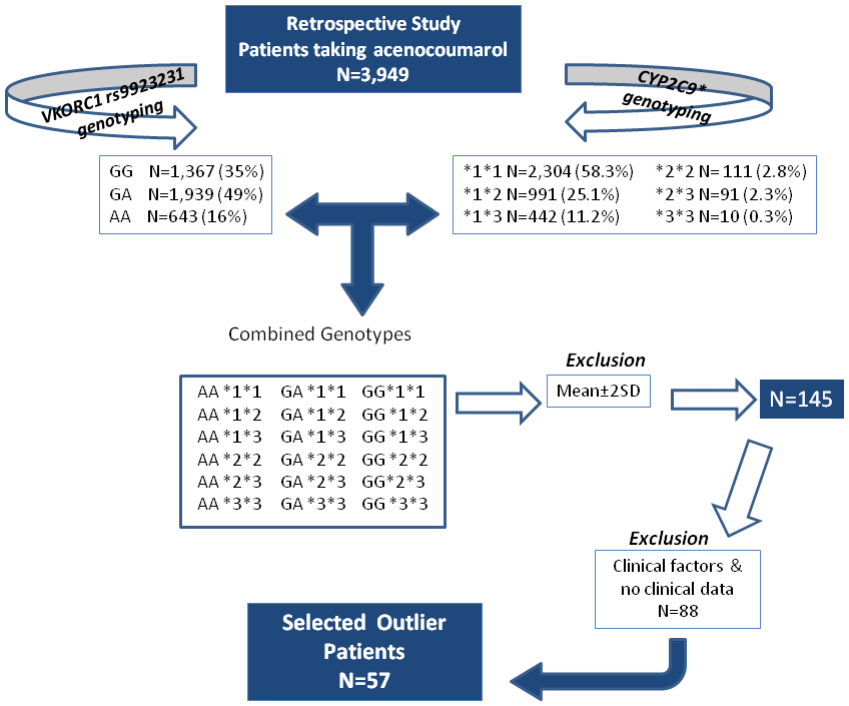
Study design: selection of patients from a retrospective cohort of subjects anticogulated with acenocoumarol (N = 3949).

The study design and eligibility are recorded in Figure 1. The three main genetic determinants of acenocoumarol dose (−1639G>A *VKORC1* and the two *CYP2C9* * SNPs) were applied to define 18 genetic profiles for the whole studied population (N = 3949) (Figure 1). For each profile, we identified the average dose. Patients with dose requirements outside the mean±2SD value according to their genetic profile were identified as ¨*outlier* ¨ patients. Patients with clinical factors that explained the abnormal dose requirements, as well as those patients with no available clinical data were excluded. In the remaining patients the promoter and coding regions of *VKORC1* were sequenced.

### Genotyping

*VKORC1* SNPs rs9923231 (−1639 G>A), rs17878544 (both in the promoter) and rs7200749 (L120L), were determined by Taqman analysis using Taqman SNP Genotyping Assays C_30403261_20, C_60247472_10 and C_29057362_10, respectively (Applied Biosystems). *VKORC1* rs9934438 (1173C>T located in intron 1) was determined using FRET probes. VKORC1 rs55894764 (R12R) and the D36Y mutation were genotyped by PCR-allelic specific restriction assay using HinfI (Promega) and RsaI (Promega), respectively. CYP2C9*2 (rs1799853) and *3 (rs1057910) genotypes were determined by the Taqman Drug Metabolism Assay C_25625805_10 and C_27104892_10, respectively (Applied Biosystems). We were unable to genotype L128R even by using different Taqman probes or restriction enzymes.

All genotypes were confirmed by sequencing of randomly selected samples.

### Sequencing

The promoter region (nucleotide number from 3377 up to 3744, according to sequence accession number AY587020) as well as the coding and flanking regions of *VKORC1* gene were sequenced in 57 selected patients. Regions were amplified by PCR and sequencing was carried out in an ABI Prism 3130*xl* Avant Genetic Analyzer (Applied Biosystems) using standard protocols.

### Statistical Analysis

Comparisons between genotype groups were performed by t test and the results are expressed as mean ± SD. Correlations were analyzed by Pearson's correlation test. A multivariate stepwise linear regression was performed to evaluate the potential contribution of different polymorphisms to inter-individual variability in the stable therapeutic acenocoumarol dose. All analyses were carried out using SPSS version 15.0 software (SPSS Inc. Chicago, IL, USA). A P-value<0.05 was considered to be statistically significant.

## Results

### Selected outlier patients

The strategy that led us to select outlier patients as well as the genetic profiles defined by *VKORC1* -1639G>A and CYPC9* polymorphisms are shown in Figure 1. Eighteen profiles covering all potential combinations were identified in our sample. The mean acenocoumarol dose for *VKORC1* rs9923231 and *CYP2C9* genotypes is described in Table 1.

**Table 1:** Prevalence and acenocoumarol reference dose of combined genetic profiles of *CYP2C9* and *VKORC1* polymorphisms in whole sample (3949 patients) and in 57 outlier patients.

Profile	CYP2C9	VKORC1	Whole sample N (%)	Whole sample reference dose$	Outlier N	Outlier with dose below reference, N	Outlier with dose above reference, N
#1	*1*1	GG	782 (20)	18±7	13	1	12
#2	*1*1	GA	1132 (29)	14±5	11	1	10
#3	*1*1	AA	390 (10)	9±4	8	-	8
#4	*1*2	GG	349 (9)	17±6	6	-	6
#5	*1*2	GA	486 (12)	13±5	9	-	9
#6	*1*2	AA	156 (4)	9±3	-	-	-
#7	*1*3	GG	160 (4)	14±5	4	-	4
#8	*1*3	GA	216 (5)	10±4	2	-	2
#9	*1*3	AA	66 (2)	7±3	3	-	3
#10	*2*2	GG	37 (1)	15±4	-	-	-
#12	*2*2	GA	56 (1)	11±6	1	-	1
#13	*2*2	AA	18 (0.5)	8±3	-	-	-
#14	*2*3	GG	35 (1)	13±5	-	-	-
#15	*2*3	GA	44 (1)	9±3	-	-	-
#16	*2*3	AA	12 (0.3)	5±1	-	-	-
#17	*3*3	GG	4 (0.1)	5±2	-	-	-
#18	*3*3	GA	5 (0.13)	11±10	-	-	-
#1	*3*3	AA	1 (0.03)	6	-	-	-

Profiles were obtained by combining genotypes VKORC1 rs9923231 (−1639G>A) and CYP2C9 rs1799853 and rs1057910.

$ mg/week expressed as mean ±SD.

One hundred and forty five patients were outside the mean±2SD of their genetic profile (3.7%). For further studies, we excluded those patients with clinical factors potentially involved in the unexpected dose requirement: autoimmune diseases (N =  10); liver failure (N =  6); severe heart failure (N =  8); severe chronic obstructive pulmonary disease (N =  10); acenocoumarol interacting drugs (N =  37); new thrombotic events (N =  6); obesity (N =  2); and associated tumoral diseases (N =  5), as well as those patients with no clinical data (N =  4). Finally, 57 out of 145 were defined as outlier patients (Figure 1).

**Figure 2:**
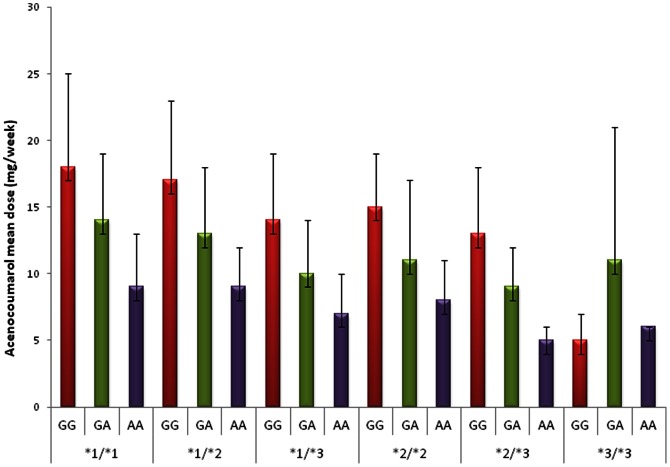
Ranking of acenocoumarol dose distribution in VKORC1 and CYP2C9 combined genetic profiles in 3949 anticoagulated patients.

Table 1 shows the mean acenocoumarol dose for each profile as well as the number of cases carrying each profile. According to the prevalence of the polymorphisms defining the profiles, the most frequent profile was represented by the *VKORC1* −1639G>A in heterozygous state plus CYP2C9*1 in homozygous state (#2, N =  1132) (Table 1 and Figure 2). In contrast, only one patient carried the profile #18 (homozygous for both *VKORC1* -1639A and CYP2C9*3). Table 1 also shows the proportion of outlier patients for each genetic profile. Most of these outlier patients took doses greater than that expected for the genetic profile (Table 1). As shown, the proportion of outlier patients was similar in all profiles (Table 1).

### *VKORC1* sequencing

Sequencing of the coding and promoter region in the 57 selected patients revealed a wild-type *VKORC1* genotype in according to reference sequence, in 43 of them (75%). *VKORC1* variants were identified in 14 outlier patients (25%), all of them with a higher acenocoumarol dose than expected (Table 2). We identified two mutations associated with high coumarin requirements and previously related with unsuccessful anticoagulation: 3 cases with D36Y [17], [18] and 1 case with L128R [19]. Additionally, there was a high prevalence of other polymorphisms: rs7200749 (L120L) in 2 patients (3.5%), rs55894764 (R12R) in 4 patients (7%), and rs17878544 a promoter variant (g.3350A>G) not found in Caucasian ancestry [20] in 5 patients (8.8%). We also identified 2 patients with no linkage disequilibrium (LD) between rs9923231 (-1639G>A) and rs9934438 (1173C>T) (3.5%) (Table 2). Additionally 2 outlier patients carried simultaneously rs17878544 and rs7200749. One patient was heterozygous for both rs55894764 and L128R (not shown).

**Table 2:** New variants of *VKORC1* identified in this study and their impact on acenocoumarol dose in whole sample (3949 patients) and in 57 outlier patients.

VKORC1 variants		Outlier N (%)	Outlier Dose*	Whole sample N (%)	Whole sample Dose*	P
D36Y	GG GT+TT	54 (94.7) 3+0 (5.3)	29±11 36±3	3935 (99.6) 14+0 (0.4)	14±6 22±9	0.004
L128R	TT TG+GG	56 (98.2) 1+0 (1.8)	29±11 36	N.D.	N.D.	N.D.
R12R (rs55894764)	GG GA+AA	53 (93.0) 4+0 (7.0)	29±11 36±6	3744 (95) 198+4 (5.2)	14±5 16±6	<0.001
L120L (rs7200749)	CC CT+TT	55 (96.5) 2+0 (3.5)	29±11 29±0.6	3861 (97.8) 85+3 (2.2)	14±6 16±7	0.001
g. 3350A>G (rs17878544)	AA AG+GG	52 (91.2) 5 (8.8)	28±10 40±13	3782(95.8) 161+5 (4.2)	14±6 15±7	0.002
LD rs2323991/ rs9934438	Yes No	55 (96.5) 2 (3.5)	30±11 14±11	3943 (99.9) 6 (0.2)	14±6 14±5	0.813

* mean ±SD of acenocoumarol expressed as mg/week. P value is calculated by t test to compare acenocoumarol dose depending on genotypes in the whole sample. One sample is missed for genotyping rs17878544 and three for R12R.

### *VKORC1* genotyping in the whole sample

These findings encouraged us to test for the prevalence of these *VKORC1* variants in the whole cohort of 3949 anticoagulated patients (Table 2).

Our findings confirmed that rs55894764 (R12R) is a common polymorphism in Caucasians with a minor allelic frequency (MAF) of 5.2% (Table 2). Interestingly, carriers seem to have higher requirements of acenocoumarol than non-carriers (14 vs 16 mg/week; p<0.001) (Table 2). On the other hand, D36Y variant was found in 14 patients (0.4%), all in heterozygous state, and this finding was also associated with a higher acenocoumarol dose (22 mg/week carriers vs 14 mg/week non-carriers; p = 0.004) (Table 2).

rs7200749 (L120L) was also a common polymorphism in our population (MAF = 2.3%), and was significantly associated with an increased dose of anticoagulant (carriers 16 vs non-carriers 14 mg/week; p = 0.001) (Table 2).

rs17878544, located in the promoter region of *VKORC1,* had a frequency of 4% in our population, with a significant relationship between dose and genotype. Thus, AA carriers needed a slightly lower acenocoumarol dose than G carriers (14 *vs* 15 mg/week respectively; p = 0.002) (Table 2).

Finally, loss of LD between rs9923231and rs9934438 was found in 6 patients (0.15%) (r2 = 0.98, D´ = 0.99)(Table 2). These data were very similar to those previously described in the bibliography [21]. The clinical data as well as the genetic profile of these 6 patients are shown in Table 3.

**Table 3:** Clinical characteristics and genetic profiles of 6 patients who lost the LD between *VKORC1* polymorphisms rs2323991 and rs9934438.

Patient	Age	Sex	Diagnostic	rs9923231	rs9934438	CYP2C9	Dose#
A	65	Female	PE	GG	CT	*1*1	19
B	70	Female	AF	GA	CC	*1*2	18
C	82	Female	AF	GA	TT	*1*1	5
D	78	Female	AF	GA	TT	*1*1	9
E	48	Male	AF	AA	CT	*1*1	24
F	65	Female	DVT	AA	CT	*1*2	12

#Dose as mg of acenocoumarol per week PE: pulmonary embolism; AF: atrial fibrillation; DVT: deep venous thrombosis

When the LD was analyzed among all SNPs found in this study, *i.e.,* rs55894764, rs7200749, rs17878544 and rs9923231, only rs17878544 was in LD with rs7200749 (r^2^ = 0.53, D´ = 0.99).

Our data showed that the VKORC1 changes were much more frequent in the outlier group than in the whole sample. Thus, Table 2 describes the differences of frequencies of the new VKORC1 variants between the outlier group and the whole sample.

### Additive regression model to predict the acenocoumarol dose

We further performed a multivariate linear regression model by including thefactors (both demographic and genetic) that resulted statistically significant in the univariate analysis. The results obtained for the multivariate linear regression model showed that, among demographical factors, only age had a significant effect on acenocoumarol dose. Thus, together with *VKORC1* rs9923231 and *CYP2C9* genotypes, the model yielded a R^2^ =  0.391 (Table 4). Interestingly, two variants identified in this study, D36Y and rs55894764, were also significantly associated with the requirement for acenocoumarol (beta coefficient 0.061and 0.034, respectively) (Table 4). However, their global contributions to dose prediction were small (from 39% to 40%).

**Table 4:** Summary of the additive regression model (stepwise method): predictor contribution to steady therapeutic dose of acenocumarol in 3949 anticoagulated patients.

	R^2^	P	ß coefficient (p)
−1639 G>A (rs9923231)	0.210	<0.001	−0.461 (<0.001)
*plus* Age	0.340	<0.001	−0.364 (<0.001)
*plus* CYP2C9	0.391	<0.001	−0.216 (<0.001)
*plus* D36Y	0.395	<0.001	−0.061 (<0.001)
*plus* R12R (rs55894764)	0.400	<0.001	0.034 (0.006)

## Discussion

Several mathematical models exist that combine both clinical and genetic data to predict the required dose of coumarins, to improve the dosing forecast in patients starting oral anticoagulant therapy [10]. The efforts of many cooperative groups of researchers have led to algorithms with r^2^ ∼50% [7], [15]. This means that a not negligible proportion of patients will still be outside the range of the best forecast models. All these data have clearly been recovered in the Clinical Pharmacogenetics Implementation Consortium published guidelines for warfarin dosing [22]. Our aim here was to identify new genetic factors that might contribute to the patient´s requirements of acenocoumarol.

The retrospective study of 3949 consecutive patients taking a steady acenocoumarol dose revealed that a small proportion of patients (N =  145, 3.7%) escaped the wide ranges of acenocoumarol dose governed by *VKORC1* and *CYP2C9* genotypes. Clinical factors potentially involved in such an abnormal response were identified in 88 of these patients (61%). The *VKORC1* gene was sequenced in the remaining patients (N =  57) in order to identify new genetic variants involved in the anticoagulant therapy. Fourteen patients (25%) had genetic alterations within *VKORC1*. For this reason, we evaluated whether these *VKORC1* genetic variations might contribute explaining the unexpected acenocoumarol dose of these patients.

We highlight the identification of two *VKORC1* variants previously linked to high coumarin doses, D36Y and L128R. The D36Y mutation has been reported as a functional mutation leading to warfarin resistance or high warfarin requirements [17], [18]. It is exceptionally common in Jews of Ethiopian origin (allele frequency 15%) and to lesser extent in Ashkenazi Jews (4%) or in Sephardi Jewish (0.6%) [23], [24]. These data clearly show that in some ethnic groups a proportion of patients would be misclassified if only the main CYP2C9 and VKORC1 variants were screened [24]. Here, in a large Caucasian cohort, the low frequency of this mutation, recently described in a small unselected patient cohort [25], is confirmed. Further studies analyzing the cost/benefit relationship of including D36Y in primary pharmacogenetic screening for initial dosing should be performed. Whatever the case, we believe that genotyping D36Y could be of benefit for those patients requiring significantly higher doses than that determined by genetic and clinical algorithms. On the other hand, although we were unable to genotype L128R on a large scale this genotype has been previously described only in rare cases with warfarin resistance [19], [26]., Similarly to D36Y, therefore, it may be speculated that this mutation should only be considered in those few patients that escape the expected forecast.

Additionally, our study suggests novel associations of *VKORC1* variants potentially involved in the pharmacogenetics of acenocoumarol. Thus, rs55894764 (R12R) was originally described in wild rats trapped in anticoagulant-exposed areas and so it might be a sign of strong evolutionary selection [27]. Our findings indicated that this variation is a polymorphism in our population that is carried by 5% of Caucasian subjects.

Although its relation with *in vivo* warfarin resistance remains elusive [27], heterozygous carriers were found to have higher acenocoumarol requirements. Further studies are required to verify this potential association and, if found, it might be of interest to performed functional studies to identify the mechanism explaining why a silent polymorphism might have functional effects on VKORC1.

Another synonymous polymorphism, rs7200749 (L120L) identified in this study has been described by two groups to have different effects on warfarin dose. In Caucasian patients this SNP had no relationship to dose [28], whereas an increase in warfarin dosage was described in a South African black population [29]. Our study suggests that this polymorphism increases the requirements for acenocoumarol with a clear genotype-dose correlation. However, in the absence of homozygous subjects, phenotypic-genotypic associations are difficult to establish. To date, rs17878544 has only been described in populations with African ancestry, and is characteristic of a haplotype predictive of high warfarin dose [20]. Given the historical connections of Spain with the African/South American slave trade and the long coexistence with Muslim from neighboring African countries, the high frequency of this allele in Spanish people may indicate population affinity.

This synonymous change could have per se a functional effect [30], although we think that its potential functional effect might be explained by its LD with rs17878544 located at *VKORC1* promoter region. Further studies, including reporter approaches, are required to sustain this hypothesis.

Finally, the loss of LD between rs9923231 and rs9934438 might also contribute to explaining the small proportion of patients taking unexpectedly high acenocoumarol doses. No more alterations were found in the sequenced VKORC1 regions of these few patients. Although our data showed that, if we take into account rs9934438, the acenocoumarol steady doses are nearer those to be expected than if we take into account rs9923231, additional studies in other populations would confirm or discard this observation. Additionally, new approaches would clarify whether rs9934438 (regardless of rs9923231) has structural consequences for VKORC1 activity and/or function.

Our work has some limitations, as the significance of these findings cannot fully be understood without functional experiments showing differential protein expression. Moreover, in this work we did not genotype for other SNPs known to contribute with smaller effect to higher dose requirements, such as in the *CYP4F2* [14], *GGCX* [31] or *CALU* [32] genes. Then, we could not rule out these SNPs as contributors to higher dose requirements in the outlier group.

In summary, in a large cohort of acenocoumarol-treated patients we found novel associations of VKORC1 variants with a higher stable dose, albeit with a low effect size. Newly available strategies such as exome sequencing or the analysis of the copy number variation [33] would help to identify variations responsible for outlier anticoagulant dosing.
